# Mediterranean Diet and Red Yeast Rice Supplementation for the Management of Hyperlipidemia in Statin-Intolerant Patients with or without Type 2 Diabetes

**DOI:** 10.1155/2013/743473

**Published:** 2013-12-23

**Authors:** Sartore Giovanni, Burlina Silvia, Ragazzi Eugenio, Ferraresso Stefania, Valentini Romina, Lapolla Annunziata

**Affiliations:** ^1^Department of Medicine-DIMED, University of Padua, Via Giustiniani, 2, 35100 Padova, Italy; ^2^Department of Pharmaceutical and Pharmacological Sciences, University of Padua, Via Giustianiani, 2, 35100 Padova, Italy

## Abstract

Lipid profile could be modified by Mediterranean diet (MD) and by red yeast rice (RYR). We assessed the lipid-lowering effects of MD alone or in combination with RYR on dyslipidemic statin-intolerant subjects, with or without type 2 diabetes, for 24 weeks. We evaluated the low-density lipoprotein (LDL) cholesterol level, total cholesterol (TC), high-density lipoprotein (HDL) cholesterol, triglyceride, liver enzyme, and creatinine phosphokinase (CPK) levels. We studied 171 patients: 46 type 2 diabetic patients treated with MD alone (Group 1), 44 type 2 diabetic patients treated with MD associated with RYR (Group 2), 38 dyslipidemic patients treated with MD alone (Group 3), and 43 dyslipidemic patients treated with MD plus RYR (Group 4). The mean percentage changes in LDL cholesterol from the baseline were −7.34 ± 3.14% (*P* < 0.05) for Group 1; −21.02 ± 1.63% (*P* < 0.001) for Group 2; −12.47 ± 1.75% (*P* < 0.001) for Group 3; and −22 ± 2.19% (*P* < 0.001) for Group 4 with significant intergroup difference (Group 1 versus Group 2, *P* < 0.001; Group 3 versus Group 4, *P* > 0.05). No significant increase in AST, ALT, and CPK levels was observed in all groups. Our results indicate that MD alone is effective in reducing LDL cholesterol levels in statin-intolerant patients with a presumably low cardiovascular risk, but associating MD with the administration of RYR improves patients' LDL cholesterol levels more, and in patients with type 2 diabetes.

## 1. Introduction

The prevalence of metabolic syndromes (MS) and the associated cardiovascular diseases (CVDs) is increasing rapidly around the world. Lifestyle measures, including dietary changes and physical activity, play a crucial role in preventing these conditions, and the National Cholesterol Education Program Adult Treatment Panel III (NCEP ATP III) has already suggested dietary intervention to contain this epidemic [1].

Cardiovascular risk factors in MS could be modified by dietary interventions. The Mediterranean diet (MD) is characterized by a high consumption of monounsaturated fatty acids (primarily from olive oil) and a daily consumption of fruit, vegetables, whole-grain cereals, and low-fat dairy products; weekly consumption of fish, poultry, tree nuts, and legumes; a relatively low consumption of red meats (approximately twice a month) [2]. The beneficial effect of the MD on all-cause mortality, CVD, and cancer, as well as on obesity and type 2 diabetes, has already been reported, based on the results of many epidemiological studies and clinical trials [3–5]. The results of a meta-analysis revealed the protective role of the MD on components of MS such as waist circumference, high-density lipoprotein cholesterol, triglycerides, systolic and diastolic blood pressure, and blood glucose levels [6]. However, acceptable total cholesterol (TC) or LDL-C levels are difficult to be achieved with lifestyle changes alone in order to significantly reduce the cardiovascular risk.

Some studies have shown that red yeast rice (RYR) reduces low-density lipoprotein (LDL) cholesterol levels in hypercholesterolemic patients [7, 8]. Becker et al. also demonstrated that RYR and therapeutic lifestyle changes reduced LDL cholesterol levels in dyslipidemic patients unable to tolerate statin therapy [9].

A combination of RYR extract, policosanol, berberine, folic acid, and Q_10_ coenzyme reportedly induced a significant metabolic improvement in elderly patients with dyslipidemia [10].

In nondiabetic patients with dyslipidemia, a combination of RYR, policosanol, berberine, folic acid, and Q_10_ coenzyme in addition to dietary counseling was found to amplify the effect of diet on central obesity, improve lipid profiles and blood pressure, and reduce the incidence of MS [11].

To our knowledge, little is known about the efficacy of RYR extract in patients with type 2 diabetes. In particular, no data are available on the effect of RYR supplementation combined with a Mediterranean diet on the lipid profiles of such patients. Hence, this randomized, parallel-group controlled study lasting six months to investigate whether adding a combination of RYR extract, artichoke extract, resveratrol, chrome, folic acid, and coenzyme Q_10_ (Redulip, For Farma srl) to the Mediterranean diet could improve the lipid profile of dyslipidemic patients with and without type 2 diabetes.

## 2. Methods

This study was designed as a controlled, randomized, parallel-group study and complied with the content of the Helsinki Declaration. The Local Institutional Review Board approved the study protocol and all participants provided written informed consent.

We studied consecutive patients attending our outpatient clinic from January to October 2010. Patients were included in the study if they had total cholesterol levels higher than 200 mg/dL and/or low-density lipoprotein (LDL) cholesterol levels higher than 130 mg/dL and a cardiovascular risk (as assessed according to the Progetto Cuore) of ≤10% for dyslipidemic patients and ≤15% for diabetic patients [12] and if they had discontinued at least one statin because of myalgias with a resolution of muscle pain when the medication was discontinued. The exclusion criteria were as follows: the presence of intima media thickness or carotid plaques on echo-color Doppler B-mode images obtained with a high-resolution imaging system (always by the same operator); use of statins up to 6 months before the study; a history of secondary dyslipidemia, except for diabetes; abnormal baseline laboratory values (a CPK level >500 U/L; AST or ALT levels >1.5 times the normal upper limit). Current and former smokers and pregnant or breast-feeding women were also ruled out.

The sample of 171 eligible participants included 90 type 2 diabetic patients with dyslipidemia and 81 dyslipidemic patients without type 2 diabetes.

At the baseline visit, we used 24-hour recall, a self-reporting method for collecting data on eating behavior and measuring energy intake by means of structured interviews, as described elsewhere [13]. A Mediterranean style dietary pattern, as previously reported [2], was recommended to all patients to improve their lipid profiles. In particular, the main goals of these recommendations were a calorie intake (between 1500 and 1800 calories) tailored to reach and/or maintain a desirable body weight, a fat intake amounting to less than 30% of the total calories (15–20% of monounsaturated fatty acids, primarily from olive oil), and a carbohydrate intake of 55–60% in dyslipidemic patients and 45–55% in diabetic patients. Patients were randomly assigned to four groups. Group 1 consisted of 46 type 2 diabetic patients treated with MD alone; Group 2 consisted of 44 type 2 diabetic patients treated with the MD plus a nutraceutical-combined pill (NCP) containing RYR extract 200 mg (equivalent to 3 mg monacolin K), artichoke extract 400 mg, resveratrol 15 mg, chromium 200 mcg, folic acid 200 mcg, and coenzyme Q_10_ 10 mg (Redulip, For Farma srl); Group 3 consisted of 38 dyslipidemic patients treated with the MD alone; Group 4 consisted of 43 dyslipidemic patients treated with the MD plus the NCP. Adherence to the medication was ascertained by means of pill counts on the study medication returned at follow-up visit.

The primary outcome was the low-density lipoprotein (LDL) cholesterol level measured at the baseline and after 24 weeks. Secondary outcomes included total cholesterol (TC), high-density lipoprotein (HDL) cholesterol, triglyceride, liver enzyme, and creatinine phosphokinase (CPK) levels.

Patients attended follow-up visits after 24 weeks of treatment. None of the patients dropped out of the study.

At each visit, all patients were assessed in terms of body mass index (BMI), diastolic and systolic blood pressure (measured with patients being seated, using a standard sphygmomanometer), and waist circumference (midway between the lowest rib and the iliac crest).

A fasting blood sample was obtained at the baseline and at week 24 to measure LDL cholesterol, TC, HDL cholesterol, triglyceride, CPK, aspartate aminotransferase (AST), and alanine aminotransferase (ALT) levels. All analyses were performed at the laboratory of the University Hospital of Padua, Italy.

At weeks 24, patients' dietary compliance was assessed using the 24-hour recall method [12] and an analysis of the food diaries completed weekly by the patients.

### 2.1. Statistical Analysis

Values are expressed as mean ± SD or SEM. Data were normalized according to each patient's baseline situation by calculating the difference (after-before) at the two follow-up times considered (24 weeks after starting the study) and expressing the value as a percentage of the parameters at the baseline. Negative values therefore indicate the effective percentage decrease in the parameter following the treatment. ANOVA followed by the *post hoc* test was used to identify statistical differences between the groups at the two different follow-up times. The statistical significance of the differences (after-before) induced by the treatment within each group of patients was tested with Student's *t*-test for paired data. Differences were considered statistically significant when *P* < 0.05 (two-tailed test).

## 3. Results

### 3.1. Baseline Characteristics

Table 1 shows the clinical characteristics of the four groups of patients at the baseline. Groups 1 and 2 differed significantly only in terms of BMI and systolic and diastolic blood pressure; Groups 3 and 4 differed significantly in terms of BMI, body weight waist circumference, and ALT levels. As regards the lipid profile, Groups 1 and 2 did not differ significantly, but Groups 3 and 4 differed significantly in terms of TC, HDL cholesterol, LDL cholesterol, and triglycerides. In particular, Group 4 had higher levels of TC (*P* < 0.001), higher levels of LDL cholesterol (*P* < 0.001), higher levels of HDL cholesterol (*P* < 0.001), and lower levels of triglycerides (*P* < 0.05).

### 3.2. Changes in Clinical Characteristics

Table 2 shows the normalized changes in the parameters after 24 weeks of treatment. Only the dyslipidemic patients treated with MD alone (Groups 3) experienced a significant reduction in body weight, BMI, and waist circumference from the baseline to week 24; the mean percentage change in these patients' BMI was −3.89 ± 0.92% (*P* < 0.001), and as regards waist circumference the reduction was −2.42 ± 0.82 cm (*P* < 0.01). All the patients kept to a Mediterranean-style diet for all 24 weeks.

### 3.3. Primary Outcome Measures

Table 2 shows the differences in the parameters in the four groups after 24 weeks of treatment. It is worth noting that the changes observed with the different treatments were significant and comparable between the groups, independently from the within-group differences at the baseline, since all the results were expressed as normalized values, taking the baseline condition of each patient for reference in the calculation. This normalization was also considered to evaluate and compare the effectiveness of the treatments appropriately.

A significant drop in TC was seen in all groups of patients. In particular, the average reduction in TC 24 weeks after the baseline was −4.65 ± 1.92% in Group 1 (*P* < 0.05), −15.45 ± 1.26% in Group 2 (*P* < 0.001), −11.96 ± 1.43% in Group 3 (*P* < 0.001), and −16.94 ± 1.51% in Group 4 (*P* < 0.001), with significant between-group differences (Group 1 versus Group 2, *P* < 0.001; Group 3 versus Group 4, *P* > 0.05).

The mean percentage changes in LDL cholesterol levels from the baseline were −7.34 ± 3.14 (*P* < 0.05) for Group 1, they were −21.02 ± 1.63% (*P* < 0.001) for Group 2, they were −12.47 ± 1.75% (*P* < 0.001) for Group 3, and they were −22 ± 2.19% (*P* < 0.001) for Group 4. The mean percentage change in LDL levels differed significantly between Groups 1 and 2 (*P* < 0.001), and between Groups 3 and 4 (*P* < 0.01).

We found no significant differences in terms of HDL cholesterol and triglycerides levels in any of the groups (Table 2).

### 3.4. Safety Data

Regarding the safety of treatment with NCP, there was no significant increase in CPK or liver-associated enzyme levels in any of the patients after 24 weeks of treatment. None of the patients discontinued the treatment with NCP and no side effects were observed. As regards liver function, we observed after 24 weeks of treatment a significant drop in AST and ALT levels in type 2 diabetic patients (Groups 1 and 2) from the baseline. In particular, we observed in Group 1 a decline in AST levels of −6.48 ± 2.83% (*P* < 0.05) and in ALT levels of −6.60 ± 2.58% (*P* < 0.05); in Group 2 we observed a decline in AST levels of −7.52 ± 3.31% (*P* < 0.05) and in ALT levels of −7.12 ± 2.96% (*P* < 0.05).

## 4. Discussion

This controlled, randomized, parallel-group study demonstrated that, in statin-intolerant patients, associating a MD with a combination of nutraceuticals (NUTs) based on red yeast rice extract can significantly improve dyslipidemic patients' lipid profiles by comparison with diet alone.

The effects of MD on lipid profile and in protecting against cardiovascular risks are well known.

In a recent meta-analysis, MD was also found associated with a lower risk of MS; in particular, several studies showed the beneficial role of MD on HDL cholesterol and triglyceride levels [6]. This meta-analysis considered studies on the general population, type 2 diabetic patients, overweight subjects, and hypercholesterolemic patients. Several randomized, controlled trials have also demonstrated that MD reduces total cholesterol and LDL cholesterol levels by 5–15% and increases HDL cholesterol levels by 3–15% [14, 15].

In agreement with previous studies, our findings showed that adherence to MD alone significantly reduced BMI, waist circumference, TC, and LDL cholesterol levels in overweight dyslipidemic patients without type 2 diabetes. The beneficial effects of MD on BMI and waist circumference were not seen in type 2 diabetic patients. These findings are consistent with other reports of weight loss programs proving less effective in overweight and obese diabetic patients [16–18]. On the other hand, we observed a significant decline in liver enzyme concentrations in type 2 diabetic patients treated with MD alone or in combination with NCP. These data are in agreement with a previous study that demonstrated that the adherence to a diet similar to MD can improve the liver function in MS subjects [19]. The treatment with RYR did not affect the beneficial effect of MD.

Our results also indicate that associating a combination of NUTs with MD can add to the lipid-lowering effect of MD, in particular on LDL cholesterol, in terms of a 10% improvement in dyslipidemic patients.

A previous study demonstrated the effect of a combination of red yeast rice extract and berberine (an isoquinoline alkaloid found in plants of the genus *Berberis *and *Coptis, *with neuroprotective and antiatherosclerotic actions [20]) associated with dietary restrictions on lipid profiles after 16 weeks of treatment, globally achieving a 23.5% reduction in LDL cholesterol levels [11]. Our present investigation is the first to be conducted on a combination of NUTs associated with MD and administered for 24 weeks. The combination of NUTs used in our study contained no berberine, which has a lipid-lowering effect in its own right, by upregulating LDL cholesterol receptors on the liver cell surface [21]. It is worth emphasizing that our results relate exclusively to the hypolipidemic effect of RYR that contains monacolin K, an inhibitor of 3-hydroxy-3-methylglutaryl-coenzyme A reductase [22], and sterols, compounds that are known to block cholesterol absorption in the gut [23, 24], with a synergic effect on lipoproteins.

Associating a combination of NUTs with a Mediterranean-style diet could prove a valuable therapeutic option for dyslipidemic statin-intolerant patients at low cardiovascular risk without excessively high LDL cholesterol levels.

The composition of the NUTs used in some previous studies varied considerably, particularly in terms of the concentration of monacolin K, which ranged from 9.6 mg to 3 mg. The dose of monacolin K in the NUTs used in our study was 3 mg. Becker et al. [9] obtained the same reduction in LDL cholesterol levels as us, but with twice as much monacolin K (6 mg). Our findings therefore suggest that a lower dose of monacolin K (about 3 mg/day) may suffice to achieve an evident therapeutic effect.

We recorded none of the adverse effects described elsewhere in patients treated with red yeast rice [25–27], probably because of the lower dose of monacolin K used in our study. Further studies are needed, however, to investigate this issue over a longer period of treatment.

To our knowledge, ours is the first study to show that associating MD with NUTs can improve, in terms of 21%, LDL cholesterol levels of type 2 diabetic statin-intolerant patients. It is often difficult to obtain a normalization of TC, or even of LDL cholesterol, with dietary restrictions alone in diabetic patients, so adding a NUT based on red yeast rice might be a good therapeutic option for type 2 diabetic patients, at low cardiovascular risk with no evidence of vascular damage or other complications, who have previous statin intolerance. The reduction in LDL cholesterol levels achieved with MD plus NUTs was similar to the reduction obtained using statins, as already reported in dyslipidemic patients with statin intolerance [7].

The limitation of this study is the small size of the sample studied; further studies on larger samples will be needed to confirm the validity of this patient management approach, particularly in cases of type 2 diabetes.

## 5. Conclusions

Despite its limitations, this study provides useful new insight into the nutraceutical/dietary treatment of lipid profiles, even in patients with type 2 diabetes. Our results indicate that MD counseling alone is effective in reducing LDL cholesterol levels in moderately hypercholesterolemic patients with a presumably low cardiovascular risk, but associating MD with the administration of RYR improved patients' lipid profiles considerably more, also in patients with type 2 diabetes with statin intolerance.

## Figures and Tables

**Table 1:** Baseline anthropometric and haematochemical parameters in the four groups of patients. Data are the mean ± standard deviation (SD).

	Group 1 Diabetic patients/diet (*n* = 46)	Group 2 Diabetic patients/diet + red rice (*n* = 44)	Group 3 Dyslipidemic patients/diet (*n* = 38)	Group 4 Dyslipidemic patients/diet + red rice (*n* = 43)	*P*	
					Group 1 versus 2	Group 3 versus 4
Gender (M/F)	19/27	18/26	20/18	14/29	n.s.	n.s.
Age (yrs)	53.28 ± 10.41	54.73 ± 11.40	52.37 ± 9.83	51.28 ± 9.68	n.s	n.s.
Height (m)	1.64 ± 0.09	1.66 ± 0.11	1.67 ± 0.08	1.66 ± 0.09	n.s.	n.s.
Body weight (kg)	84.68 ± 10.25	82.10 ± 17.26	77.95 ± 14.15	70.25 ± 12.01	n.s.	●
BMI	31.57 ± 3.73	29.70 ± 5.02	27.77 ± 4.26	25.36 ± 3.16	●	●●
Waist (cm)	98.64 ± 11.47	96.75 ± 12.69	94.61 ± 12.07	86.70 ± 10.43	n.s	●●
TC (mg/dL)	231.13 ± 22.01	234.80 ± 19.09	251.37 ± 32.76	275.81 ± 32.20	n.s.	●●●
LDL (mg/dL)	149.07 ± 21.95	148.72 ± 20.38	168.23 ± 31.70	188.98 ± 28.79	n.s.	●●●
HDL (mg/dL)	54.15 ± 13.17	60.00 ± 15.46	49.66 ± 12.50	61.26 ± 16.94	n.s.	●●●
TG (mg/dL)	139.52 ± 53.69	130.36 ± 58.32	167.42 ± 100.75	127.91 ± 65.82	n.s.	●
CPK (ng/mL)	112.47 ± 40.68	110.59 ± 35.74	114.03 ± 40.95	114.23 ± 46.57	n.s	n.s.
AST (mU/mL)	27.37 ± 17.34	29.18 ± 19.92	23.03 ± 7.83	22.44 ± 6.14	n.s	n.s.
ALT (mU/mL)	30.74 ± 18.58	32.41 ± 17.62	32.95 ± 23.89	22.09 ± 7.37	n.s	●●
GGT (mU/mL)	26.53 ± 19.45	28.43 ± 20.17	29.55 ± 28.17	22.05 ± 10.89	n.s	n.s.
Systolic BP (mmHg)	142.80 ± 15.00	134.05 ± 14.39	130.53 ± 17.00	124.77 ± 15.08	●●	n.s.
Diastolic BP (mmHg)	86.07 ± 10.74	81.52 ± 8.15	81.18 ± 10.36	77.56 ± 8.89	●	n.s.

To assess statistical differences between groups, ANOVA followed by *post hoc* test was used for continuous data; for frequency data, *χ* ^2^ test was used.

●●●: *P* < 0.001; ●●: *P* < 0.01; ●: *P* < 0.05; n.s.: not significant.

Abbreviations: BMI: body mass index; TC: total cholesterol; LDL: low-density lipoprotein; HDL: high-density lipoprotein; TG: triglyceride; CPK: creatinine phosphokinase; AST: aspartate aminotransferase; ALT: alanine aminotransferase; GGT: gamma glutamyl transferase; BP: blood pressure.

**Table 2:** Differences (Δ) (after − before), expressed as percentage of basal value, in parameters observed in the four groups of patients, following 24 weeks of treatment.

Difference (after − before) % of basal at 6 months	Group 1 Diabetic patients diet (n = 46)		Group 2 Diabetic patients diet + red rice (*n* = 44)		Group 3 Dyslipidemic patients diet (*n* = 38)		Group 4 Dyslipidemic patients diet + red rice (*n* = 43)		P	
									Group 1 versus 2	Group 3 versus 4
Δ-Body weight % (kg)	0.89	±0.83	−0.86	±0.55	−3.89	±0.92^†††^	−0.87	±0.51	n.s.	●●
Δ-BMI %	0.89	±0.83	−0.86	±0.55	−3.89	±0.92^†††^	−0.87	±0.51	n.s.	●●
Δ-Waist % (cm)	−0.89	±0.32	−0.93	±0.44	−2.42	±0.82^††^	−0.98	±0.55	n.s.	n.s.
Δ-TC % (mg/dL)	−4.65	±1.92^†^	−15.45	±1.26^†††^	−11.96	±1.43^†††^	−16.94	±1.51^†††^	●●●	●
Δ-LDL % (mg/dL)	−7.34	±3.14^†^	−21.02	±1.63^†††^	−12.47	±1.75^†††^	−22.00	±2.19^†††^	●●●	●●
Δ-HDL % (mg/dL)	2.87	±2.38	−2.93	±1.72	−0.11	±2.67	−1.94	±1.53	●	n.s.
Δ-TG % (mg/dL)	2.30	±4.32	−3.77	±4.33	−7.24	±7.53	−7.52	±3.83	n.s.	n.s.
Δ-CPK % (ng/mL)	−2.84	±1.32	−3.51	±1.84	−1.15	±1.36	2.14	±5.62	n.s.	n.s.
Δ-AST % (mU/mL)	−6.48	±2.83^†^	−7.52	±3.31^†^	0.52	±4.52	−0.78	±2.40	n.s.	n.s.
Δ-ALT % (mU/mL)	−6.60	±2.58^†^	−7.12	±2.96^†^	−12.37	±6.02^†^	1.60	±3.30	n.s.	●
Δ-GGT % (mU/mL)	8.47	±15.74	10.30	±18.64	−6.89	±4.39	−1.48	±4.67	n.s	n.s.
Δ-Systolic BP % (mmHg)	3.42	±2.13	−2.06	±1.45	−0.47	±1.83	1.51	±1.75	●	n.s.
Δ-Diastolic BP % (mmHg)	3.03	±2.80	−0.41	±1.33	−0.23	±1.75	0.49	±1.56	n.s.	n.s.

Statistical significance for difference (after − before); treatment within each group of patients was checked with Student's *t*-test for paired data. ^†††^ *P* < 0.001; ^††^ *P* < 0.01; ^†^ *P* < 0.05.

To assess statistical differences between groups, ANOVA followed by *post hoc* test was used. ●●●: *P* < 0.001; ●●: *P* < 0.01; ●: *P* < 0.05; n.s.: not significant.

Abbreviations: BMI: body mass index; TC: total cholesterol; LDL: low-density lipoprotein; HDL: high-density lipoprotein; TG: triglyceride; CPK: creatinine phosphokinase; AST: aspartate aminotransferase; ALT: alanine aminotransferase; GGT: gamma glutamyl transferase; BP: blood pressure.

Data are the mean ± standard error of the mean (SEM). Negative values indicate a decrease of the parameter after respective treatment.
